# Intact pulse cotyledon cell diet improves systemic growth via the somatotropic axis in malnourished mice

**DOI:** 10.1002/imo2.70085

**Published:** 2026-02-25

**Authors:** Yumeng Yan, Zhuqing Xie, Zhipeng Gu, Zhongxin Li, Qiang Huang, Bin Zhang

**Affiliations:** ^1^ School of Food Science and Engineering, National Engineering Research Center of Wheat and Corn Further Processing, Guangdong Province Key Laboratory for Green Processing of Natural Products and Product Safety South China University of Technology Guangzhou China

**Keywords:** gut microbiota, malnutrition, pulse intact cell, SCFA, somatotropic axis

## Abstract

Malnutrition, often associated with dysbiosis of the gut microbiota, requires effective dietary interventions. This study evaluated the effects of diets containing intact pulse cotyledon cells (MAL‐IC diet) or pulse flour (MAL‐PF diet) within a malnourished mouse model. The MAL‐IC diet significantly improved systemic growth compared with the malnutrition (MAL: low‐fat [2%], low‐protein [6.4%]) diet group, outperforming the MAL‐PF diet. Additionally, the MAL‐IC diet restructured the gut microbiota and metabolome, increasing the relative abundance of carbohydrate‐utilizing bacteria and short‐chain fatty acids (SCFAs). Notably, the MAL‐IC diet stimulated the somatotropic axis, thereby promoting higher insulin‐like growth factor‐1 (IGF‐1) production across multiple tissues. Integration of gene expression data revealed that SCFAs activated hepatic free fatty acid receptor 2 (FFAR2) via the gut–liver axis, further stimulating the somatotropic axis to promote growth. These results demonstrate that an intact cell‐based diet restores growth through microbiota–SCFA–endocrine interactions and propose a pulse‐enriched food strategy to address malnutrition.

## Highlights

- Intact pulsed cotyledon cells treat malnutrition more effectively than traditional flour and promote systemic growth and metabolic health in mice.
- These effects are mediated by enriching gut *Lactobacillus*, enhancing short‐chain fatty acid production, and stimulating the growth‐related somatotropic axis.
- This study underscores the importance of food matrix integrity, offering a promising whole‐food strategy for nutritional intervention.

## Graphical abstract

The MAL‐IC diet promotes growth in malnourished mice by regulating the gut microbiota—increasing the abundance of carbohydrate‐utilizing bacteria and SCFAs production. SCFAs circulate to the liver via the gut–liver axis, where they bind FFAR2 and activate the somatotropic axis, thereby stimulating GHR‐mediated GH signaling and increasing IGF‐1 and IGFBP‐3 production, ultimately promoting systemic growth.

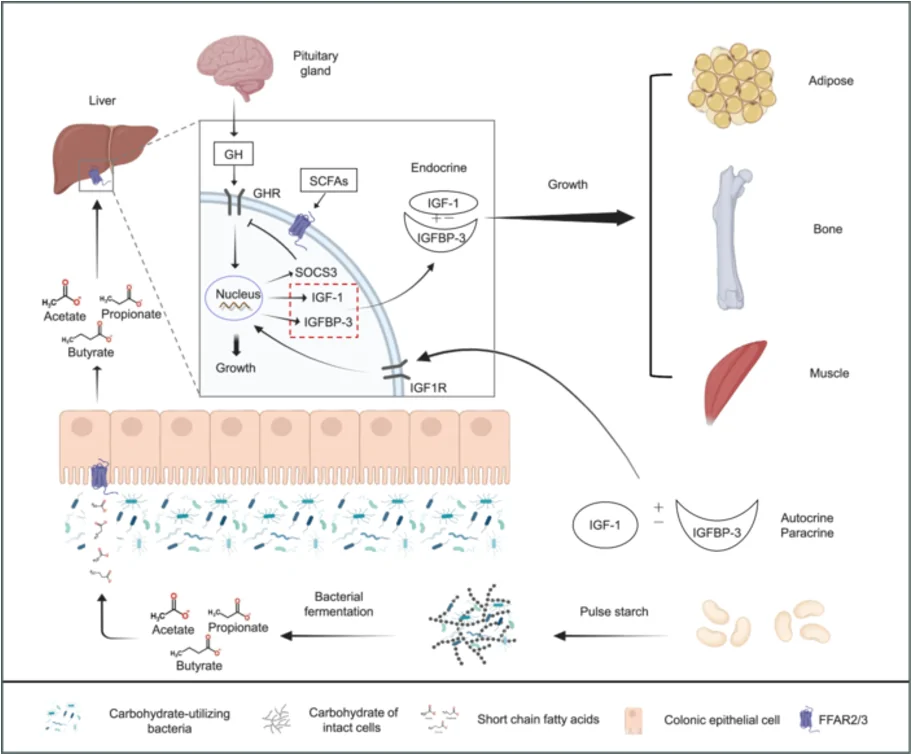

## 1 INTRODUCTION

Malnutrition represents a significant global health challenge [1], affecting over 10% of the worldwide population, with prevalence highest in low‐income regions, such as East Africa and Middle‐South Asia [2]. The development of malnutrition is driven by complex interactions between multiple factors [3]. Fecal‐oral contamination resulting from inadequate sanitation infrastructure, unsafe water supplies, and poor hygiene practices remains a primary contributor to persistent early‐life malnutrition [4, 5, 6]. Exposure to fecal pathogens disrupts gut microbiota balance and impairs nutrient absorption [7, 8]. Additional contributors include insufficient breastfeeding, weakened immunity, diarrheal diseases, and enteritis, all of which amplify susceptibility to undernutrition [9]. Current therapeutic strategies, including antibiotic treatment and energy supplementation, have shown limited long‐term effectiveness, underscoring an ongoing inability to restore metabolic homeostasis in affected individuals [10].

The composition of gut microbiota differs markedly between malnourished and healthy children [11, 12]. Affected children often exhibit immature gut microbial profiles characterized by reduced diversity [3]. The establishment and maturation of gut microbiota in newborns critically influence nutrient absorption [13], highlighting the microbiome as a key therapeutic target in childhood malnutrition [14, 15]. For example, Gehrig et al. administered either a microbiota‐directed complementary diet or a ready‐to‐use high‐energy supplementary diet (nutrient‐dense paste) to 123 moderately undernourished infants (12–18 months) living in Bangladeshi urban slums [10]. Their findings indicated that the microbiota‐directed diet improved key growth biomarkers—including skeletal development, cognitive function, and immune responses—shifting these metrics closer to the levels observed in well‐nourished pediatric populations. Further analyses revealed that dietary intervention induced beneficial reconfiguration of the gut microbiota in malnourished children [10], affirming the pivotal role of a healthy gut microbiome in supporting child growth.

Among the principal components of these microbiota‐targeted therapeutic diets, pulses might be particularly important. Calder et al. conducted a pilot study involving 58 Ugandan children with severe acute malnutrition, comparing a cowpea‐enriched regimen to a conventional diet over 4 weeks, and found that a cowpea‐flour‐based intervention increased microbial diversity and short‐chain fatty acid (SCFA) production in the gut of malnourished children [16]. These results suggest that pulse‐enriched diets can help restore gut microbiome balance and barrier integrity, thereby mitigating childhood malnutrition. Intact pulse cotyledon cells function as a resistant starch type 1 (RS1) due to their intact cell walls, which resist enzymatic digestion [17]. Numerous studies have shown that most intact pulse cotyledon cells evade gastrointestinal breakdown; their nutrients are instead fermented by colonic microbes [18, 19]. In contrast, processing pulse flour disrupts cellular architecture, leading to rapid digestion and absorption in the upper gastrointestinal tract [17]. This evidence collectively supported the hypothesis that intact pulse cotyledon cells may provide greater functional benefits than traditional pulse flours in dietary interventions for malnutrition.

In the present study, we performed a comparative analysis evaluating the differential effects of intact pulse cotyledon cells versus pulse flour within a murine model of malnutrition. We assessed growth performance, glucose–lipid homeostasis, inflammatory biomarkers, hepatic histopathology, and somatotropic axis activity to evaluate the systemic ameliorative effects in malnourished mice. Advanced methodologies, such as 16S rRNA sequencing and untargeted metabolomic profiling, were utilized to characterize gut microbiota composition and associated metabolic changes. To define the underlying mechanisms, we quantified hepatic expression of key somatotropic mediators and established functional correlations with SCFA levels using synthetic correlation analysis. Collectively, this study provides novel insights into the design of functional food ingredients and offers a foundation for evidence‐based nutritional interventions targeting malnutrition.

## 2 RESULTS AND DISCUSSION

### MAL‐IC diet improves systemic growth in malnourished mice

To assess the impact of intact pulse cotyledon cells within a malnourished mouse model (MAL‐IC diet) on malnutrition, a moderate malnutrition mouse model was established. Briefly, 3‐week‐old C57BL/6J mice were administered a protein/fat‐deficient carbohydrate‐rich diet for 4 weeks (Figure 1A). The growth parameters (i.e., body weight and length gain) of the malnourished (MAL) group were severely altered relative to the control (CON) group (Figure 1B–F). At the end of week 4, the MAL group exhibited stunted growth, evidenced by significantly lower body weight and shorter body length than the CON group (Figure 1C,D). Diminished body weight gain corresponded with a decrease in growth rate (Figure 1E), while no statistically significant difference was identified in body length growth rate between the groups (Figure 1F). Additionally, the MAL group had significantly higher food intake than the CON group over Weeks 0–4 (Figure 1G). These findings suggest that the stunted phenotype in the MAL group resulted from inadequate nutrient utilization and storage, despite sufficient or increased food consumption [20].

**Figure 1:**
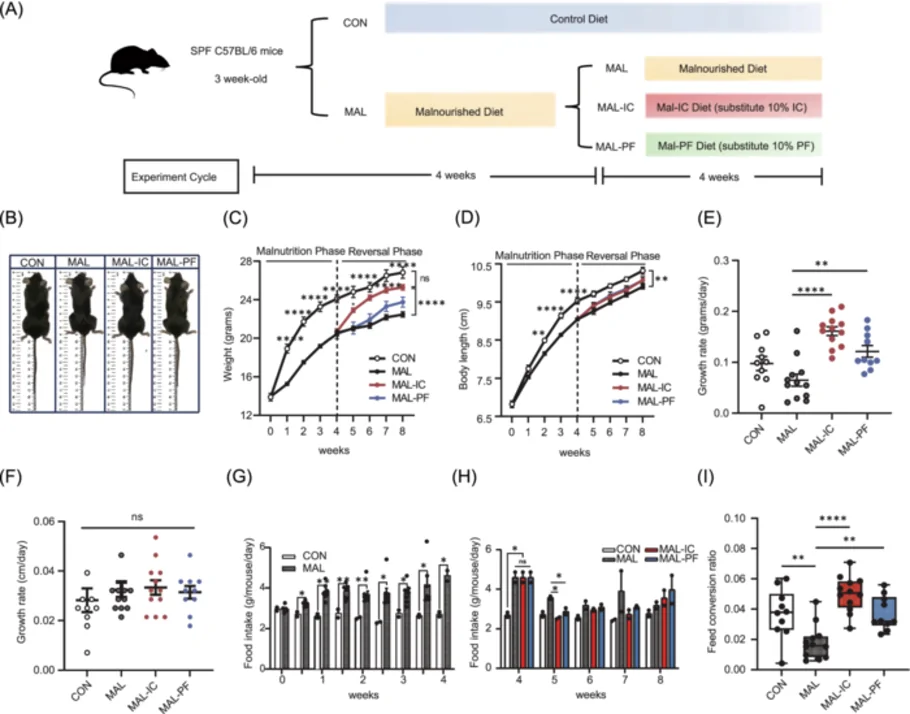
MAL‐IC diet improved systemic growth and restored the feed conversion ratio in the MAL group. (A) Experimental design. (B) Representative images of C57BL/6J mice fed with four different diets. Body weight (C), body length (D), weight growth rate (E), and length growth rate (F) of CON, MAL, MAL‐IC, and MAL‐PF (*n* = 10 per group). (G–I) Food intake during the malnutrition modeling period (G) and intervention period (H), and feed conversion ratio (I) of CON, MAL, MAL‐IC, and MAL‐PF (*n* = 10 per group). Data are presented as mean ± SEM. Data are presented as box plots showing the median (center line), 25th and 75th percentiles (box limits), and the minimum and maximum values within 1.5× interquartile range (whiskers). *p < 0.05, **p < 0.01, ***p < 0.001, ****p < 0.0001; ns, not significant. (CON, mice fed a standard diet; MAL, mice fed a malnourished diet; MAL‐IC, mice fed a malnourished diet containing intact pulse cotyledon cells; MAL‐PF, mice fed a malnourished diet containing pulse flour).

Evidence suggests that pulse‐rich dietary interventions may confer therapeutic benefits for malnutrition [21, 22]. In the current study, the MAL‐IC diet was administered to the MAL group for an additional 4 weeks, alongside a parallel group fed with a malnourished diet containing pulse flour (MAL‐PF) (Figure 1A). Although intact pulse cotyledon cells and pulse flour mainly differ in their impact on cell wall integrity—with enzymatic and mechanical treatments altering cellular structure without substantially changing overall composition, the nutrients in pulse flour are more readily accessible, potentially providing higher energy availability than intact pulse cotyledon cells. However, despite its presumably lower caloric density, intact pulse cotyledon cells alleviated malnutrition more effectively, suggesting their beneficial effect may be mediated by modulating the gut microbiota and associated metabolites, rather than energy intake alone.

At the end of week 8, the MAL‐IC group demonstrated a significant increase in body weight and corresponding growth rate compared to the MAL group (Figure 1C,E). Conversely, in the MAL‐PF group, only the body weight growth rate significantly exceeded that of the MAL group (Figure 1E). Changes in body weight among mice during Weeks 5–8 were not attributable to variations in food intake (Figure 1H). Furthermore, there were no significant differences in body length or corresponding growth rates among the four groups during Weeks 5–8 (Figure 1D,F), suggesting that neither the MAL‐IC nor MAL‐PF diets had a significant effect on bone growth.

The feed conversion ratio (FCR) is a vital metric for evaluating the efficiency of feed conversion into body mass, thereby reflecting the capacity for nutrient absorption and utilization [23]. A significant decline was detected in FCR within the MAL group during Weeks 5–8 compared with the CON group (Figure 1I). Although FCR was significantly improved in the MAL‐IC and MAL‐PF groups, the MAL‐IC diet demonstrated a greater effect, suggesting its potential to enhance systemic growth in malnourished mice.

### MAL‐IC diet enhances bone area, promotes adipose weight gain, and alleviates liver inflammation in malnourished mice

Malnutrition impairs cartilage growth and bone formation by stimulating inflammatory factors and altering gut microbiota [24, 25]. Dual‐energy X‐ray absorptiometry (DEXA), a noninvasive imaging technique, was used to measure bone parameters in mice [26]. At the end of week 8, the MAL group exhibited significantly lower bone mineral density (BMD), bone mineral content (BMC), BMC%, bone volume (BV), and bone area (BA) compared with the CON group (Figure 2A–E, Figure S1), indicating that chronic undernutrition impaired bone development, diminished bone mass, and reduced bone density. Notably, the MAL‐IC group exhibited substantial improvements over the MAL group, whereas the MAL‐PF group showed no significant difference (Figure 2E), suggesting that the MAL‐IC diet positively affected bone health in mice.

**Figure 2:**
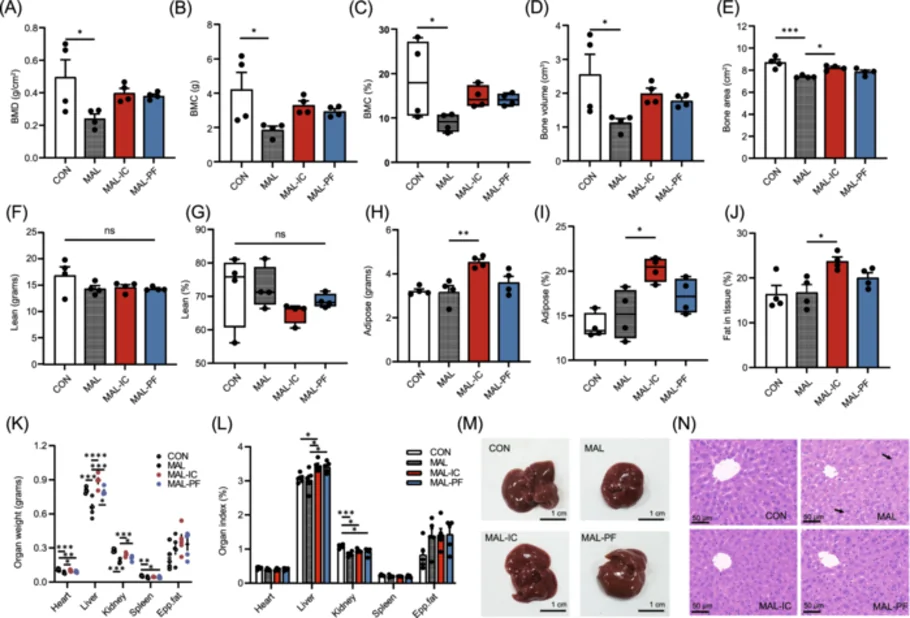
MAL‐IC diet promoted bone growth, increased the weight of adipose tissue and liver, and alleviated liver inflammation in the MAL group. (A) Bone mineral density. (B, C) Bone mineral content and its percentage. (D, E) Bone volume and bone area. (F, G) The weight and percentage of lean tissue. (H, I) The weight and percentage of adipose tissue. (J) Percentage of fat adhered to tissues in mice. (K, L) Organ weight and organ index. (M, N) Representative images of the whole liver and H&E‐stained liver sections. Malnourished livers exhibit microvesicular steatosis, as indicated by the direction of the black arrows in the figure. The data analysis was based on four mice per group. Data are presented as mean ± SEM. Data are presented as box plots showing the median (center line), 25th and 75th percentiles (box limits), and the minimum and maximum values within 1.5× interquartile range (whiskers). *p < 0.05, **p < 0.01, ***p < 0.001, ****p < 0.0001; ns, not significant. (CON, mice fed a standard diet; MAL, mice fed a malnourished diet; MAL‐IC, mice fed a malnourished diet containing intact pulse cotyledon cells; MAL‐PF, mice fed a malnourished diet containing pulse flour).

The absolute and relative weights of adipose and lean tissues contribute to overall body weight [27]. No statistically significant differences in lean weight were detected among the four groups (Figure 2F,G); however, adipose tissue measurements showed a significantly higher adipose mass in the MAL‐IC group compared with the MAL group (Figure 2H–J). These findings imply that the MAL‐IC diet may influence lipid metabolic pathways closely associated with liver function [28]. To further assess liver function, changes in organ weight and index were analyzed, as previously described [29]. A significant decrease in liver weight and organ index was observed in the MAL group compared with the other three groups (Figure 2K,L), attributed primarily to reduced glycogen and fat content in hepatocytes and interstitial adipose tissue [30]. The MAL‐IC diet substantially restored liver weight to normal levels (Figure 2K,M), unlike the MAL‐PF diet. Histological analysis also revealed pronounced steatosis in the livers of the MAL group, characterized by vacuolation and displaced cell nuclei (Figure 2N), corroborating previous reports [4, 26]. Steatosis incidence and the number of cytoplasmic vacuoles were considerably reduced in the MAL‐IC group, outperforming the MAL‐PF group (Figure 2N). Collectively, these results suggest that the MAL‐IC diet increases adipose tissue weight and mitigates liver inflammation in malnourished mice.

### MAL‐IC diet restores glucose levels and lipid metabolism, alleviates chronic inflammation, and enhances growth‐related hormone secretion in malnourished mice

Malnourished mice typically exhibit disorders of glucose metabolism [31, 32]. The fasting blood glucose concentrations in the CON group were significantly higher than those recorded in the other three groups (Figure 3A), indicating that prolonged low‐fat and low‐protein diets impair glucose homeostasis [31]. Assessing the area under the curve (AUC) further reflected the capacity for glucose regulation [33]. No significant differences were observed in the AUC values between the CON and MAL‐IC groups, suggesting that the MAL‐IC diet successfully restored glucose tolerance to normal levels in undernourished mice (Figure 3B,C).

**Figure 3:**
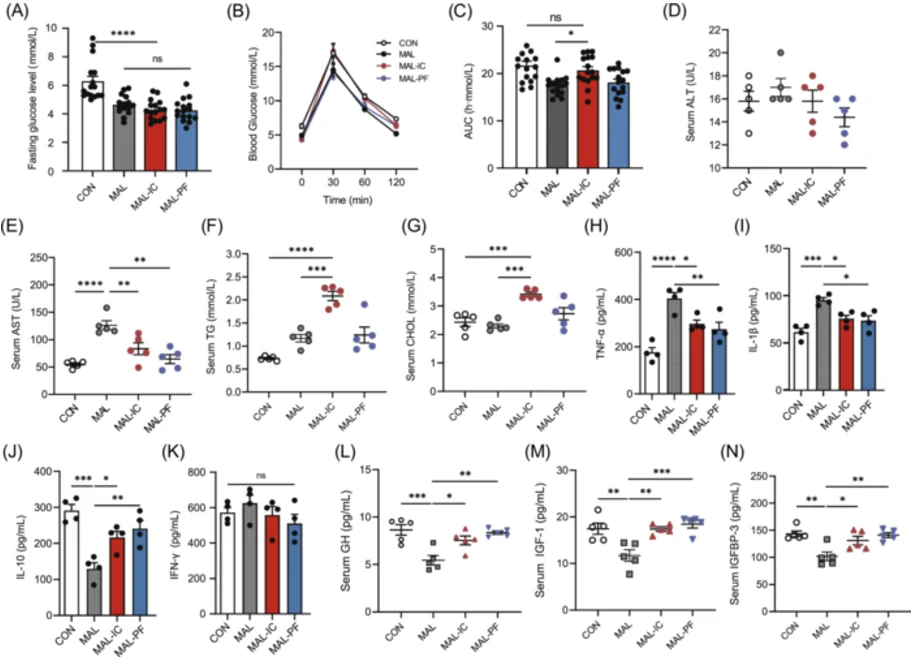
MAL‐IC diet restored the levels of glucose and lipid metabolism, stabilized chronic inflammation, and promoted growth‐related hormones secretion in the MAL group. Results of oral glucose tolerance test, including fasting glucose level (A), blood glucose level curve over time (B), and AUC (C) of CON, MAL, MAL‐IC, and MAL‐PF (*n* = 10 per group). (D–G) Serum ALT, AST, TG, and CHOL levels of CON, MAL, MAL‐IC, and MAL‐PF (*n* = 5 per group). (H–K) Serum cytokine TNF‐α, IL‐1β, IL‐10, and IFN‐γ levels of CON, MAL, MAL‐IC, and MAL‐PF (*n* = 5 per group). (L–N) Serum GH, IGF‐1, and IGFBP‐3 levels of CON, MAL, MAL‐IC, and MAL‐PF (*n* = 5 per group). Data are presented as mean ± SEM. *p < 0.05, **p < 0.01, ***p < 0.001, ****p < 0.0001; ns, not significant (CON, mice fed a standard diet; MAL, mice fed a malnourished diet; MAL‐IC, mice fed a malnourished diet containing intact pulse cotyledon cells; MAL‐PF, mice fed a malnourished diet containing pulse flour).

To further evaluate hepatic function in mice, we measured relative biochemical serum markers. Alanine aminotransferase (ALT) and aspartate aminotransferase (AST) are common indicators of liver health, with AST elevation indicating severe tissue damage and ALT serving as a sensitive marker of liver injury [34]. AST levels were markedly higher in the MAL group than in the CON group, and both MAL‐IC and MAL‐PF diets significantly reduced these enzyme levels (Figure 3D,E). These findings are consistent with histological assessments from hematoxylin and eosin (H&E)‐stained liver sections (Figure 2N).

Triglycerides (TG) are the main form of fat storage, and cholesterol (CHOL)—a key indicator of lipid metabolism [35]—was also quantified. Although significant differences were not observed in serum TG and CHOL levels between the CON and MAL groups, they were both markedly elevated in the MAL‐IC group (Figure 3F,G). Hence, MAL‐IC diet intervention may have restored lipid metabolism (Figure 3E,G) while significantly increasing fat content (Figures 2H–J, 3F) and horizontal growth (Figure 1C) in MAL mice.

Inflammatory status was assessed by quantifying serum cytokines [36] (Figure 3H–K). As anticipated, the MAL group exhibited significantly elevated pro‐inflammatory serum cytokines (i.e., tumor necrosis factor [TNF]‐α and interleukin [IL]−1β) and decreased anti‐inflammatory cytokines (IL‐10) relative to the CON group (Figure 3H–J). Conversely, both MAL‐IC and MAL‐PF groups exhibited significantly lower TNF‐α and IL‐1β levels and higher IL‐10 (Figure 3H–J), suggesting effective reversal of malnutrition‐induced chronic inflammation in mice.

Insulin‐like growth factor‐1 (IGF‐1) is a key endocrine regulator of postnatal growth and is suppressed by nutritional deficiency [37, 38]. According to the somatomedin hypothesis [39], pituitary‐derived growth hormone (GH) stimulates hepatic IGF‐1 synthesis, which complexes with insulin‐like growth factor‐binding protein‐3 (IGFBP‐3) and promotes tissue growth in bones, muscles, and other organs [40]. Accordingly, serum levels of GH, IGF‐1, and IGFBP‐3 were measured and found to be significantly diminished in MAL mice compared to the CON group, consistent with previous observations [31] (Figure 3L–N). Moreover, both MAL‐IC and MAL‐PF diets significantly elevated serum levels compared with the MAL group (Figure 3L–N), underscoring the therapeutic potential of dietary interventions to ameliorate the adverse effects of malnutrition.

### MAL‐IC diet alters gut microbiota composition

Individuals with malnutrition exhibit notable differences in gut microbiota compared to healthy individuals, characterized by reduced microbial diversity and a high relative abundance of harmful bacteria [10, 41]. To evaluate the impact of malnutrition and pulse diet interventions on colonic microbiota in mice, 16S rRNA gene sequencing was performed on fecal samples from the four experimental groups after 8 weeks. Both the MAL‐IC and MAL‐PF groups exhibited significant increases in Chao 1, observed OTUs, and Shannon indices compared to the MAL group (Figure 4A). Moreover, community similarity was assessed using β‐diversity metrics (Bray–Curtis and Unifrac‐unweighted distances) [42, 43, 44]. These analyses revealed that the resident gut microbiota of the CON group differed substantially from those of the other three groups (Figure 4B), indicating that prolonged exposure to a low‐fat, low‐protein diet altered microbial community structure [4]. Similarly, diets containing intact pulse cotyledon cells or pulse flour modulated habitat diversity relative to the MAL diet.

**Figure 4:**
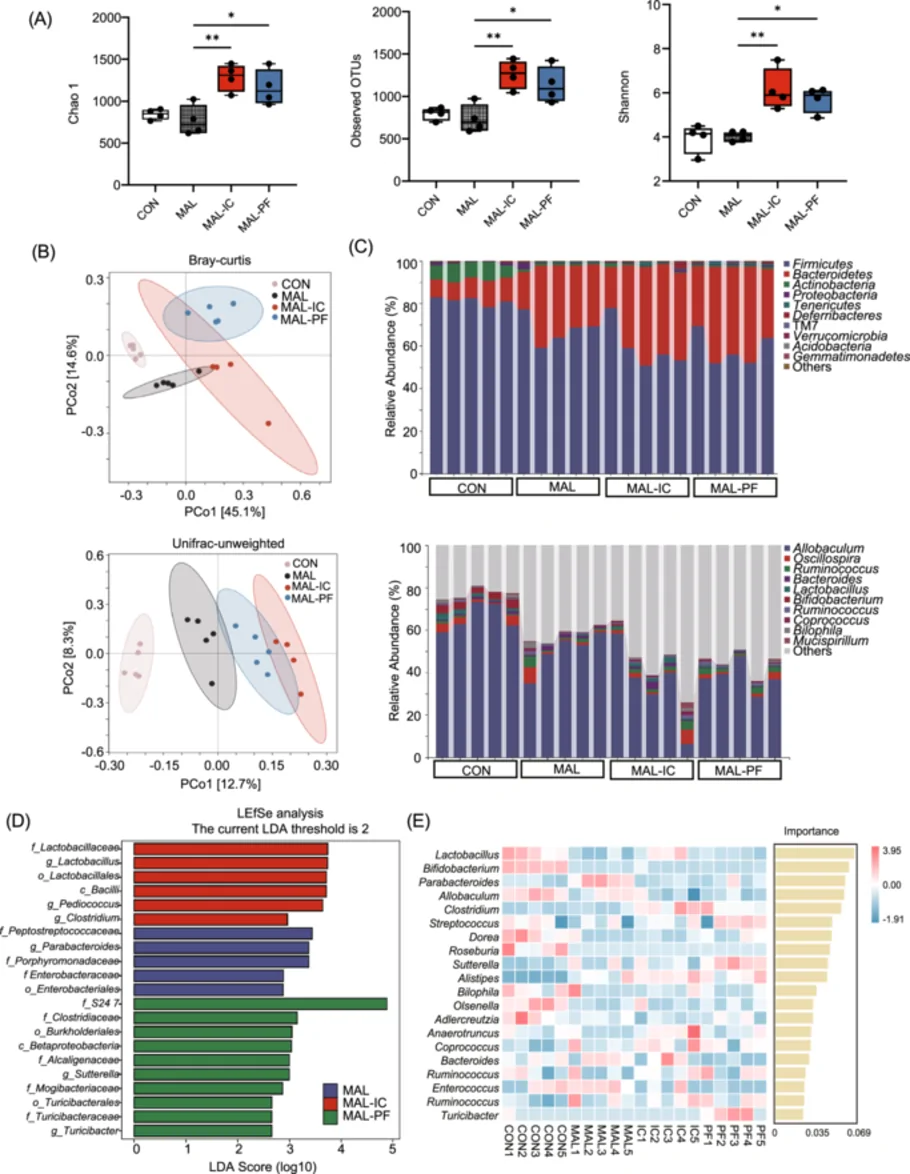
Pulse dietary intervention altered gut microbiota composition, and the MAL‐IC diet promoted the abundance of *Lactobacillus*. (A) Measures of α‐diversity of intestinal microbiota of mice fed four different diets, including Chao 1, Observed OTUs, and Shannon indexes. (B) Bray–Curtis and Unifrac‐unweighted based PCoA analysis of CON, MAL, MAL‐IC, and MAL‐PF microbiota. Each symbol represents the value for individual mouse, and symbol color reveals different diets. (C) 16S rRNA microbiota analyses from murine individual fecal samples. Relative abundance of bacteria by phylum and genus classification at the final time point is shown. (D) LEfSe analysis reveals the differentially abundant taxa among MAL, MAL‐IC, and MAL‐PF groups. The length of the bar chart indicates the contribution level of different species, namely the LDA score, where a higher value signifies more significant differences. (E) Random forest analysis among the top 20 differentially expressed strains across groups. The vertical axis represents the names of taxonomic units at the genus level, while the bar chart indicates the importance scores of species for the classifier model. Fecal samples were collected post‐intervention, with five samples per group subjected to 16S rRNA sequencing. Data are presented as mean ± SEM. Data are presented as box plots showing the median (center line), 25th and 75th percentiles (box limits), and the minimum and maximum values within 1.5× interquartile range (whiskers). *p < 0.05, **p < 0.01, ***p < 0.001, ****p < 0.0001. (CON, mice fed a standard diet; MAL, mice fed a malnourished diet; MAL‐IC, mice fed a malnourished diet containing intact pulse cotyledon cells; MAL‐PF, mice fed a malnourished diet containing pulse flour).

To determine which microbes were responsible for changes in diversity and community structure, bacteria were classified at various phylogenetic levels. At the phylum level, the relative abundances of Firmicutes and Actinobacteria decreased, whereas those of Bacteroidetes increased in the MAL, MAL‐IC, and MAL‐PF groups compared to the CON group (Figure 4C). Additionally, the families Ruminococcaceae, Lachnospiraceae, and Muribaculaceae—with critical roles in carbohydrate fermentation [45]—were more abundant in the MAL, MAL‐IC, and MAL‐PF groups than in the CON group (Figure S2). Considering that the MAL diet is low in fat and protein and restricts nitrogen sources, it elicits a reconfiguration of the gut microbiota to sustain metabolic homeostasis, with microbial metabolism shifting primarily toward carbon metabolism [46]. Moreover, at the genus level, the relative abundances of *Allobaculum*, *Oscillospira*, *Bifidobacterium*, and *Bilophila* decreased, while those of *Ruminococcus*, *[Ruminococcus]*, and *Coprococcus* increased in the MAL, MAL‐IC, and MAL‐PF groups compared to the CON group (Figure 4C). Furthermore, *Bacteroides*, *Lactobacillus*, and *Mucispirillum* in the MAL‐IC group than in the MAL‐PF group (Figure 4C). These results were supported by Linear Discriminant Analysis Effect Size (LefSe) and random forest analyses (Figure 4D). LEfSe analysis identifies taxa with statistically significant differences in abundance among groups, whereas random forest analysis predicts and ranks the importance of features that contribute to group differentiation [47, 48]. Additionally, the CON/MAL‐IC group exhibited a significantly higher abundance of *Lactobacillus* spp. than the MAL/MAL‐PF group (Figure S3). Taken together, these results suggest that the MAL‐IC diet specifically promotes the growth of beneficial carbohydrate‐utilizing bacteria, led by *Lactobacillus* [49].

### MAL‐IC diet modulates the gut metabolome

Alterations in the microbial community invariably lead to changes in the metabolic states [50]. Untargeted metabolite analysis of fecal samples from the four groups revealed that most samples within the MAL and MAL‐PF groups clustered in the hierarchical clustering dendrogram within one category, whereas most CON and MAL‐IC group samples clustered into another distinct category (Figure 5A). This suggested that intervention with the MAL‐IC diet resulted in metabolic profiles within the MAL‐IC group that closely resembled those of the CON group (Figure 5B) [51]. Principal component analysis (PCA) and partial least squares‐discriminant analysis (PLS‐DA) of the untargeted metabolome confirmed distinct differences among the groups (Figure 5C,D), with clear separation observed between the CON and MAL groups. Additionally, overlap between the MAL‐IC and MAL‐PF groups indicated a potential convergence in metabolic patterns following these two dietary interventions.

**Figure 5:**
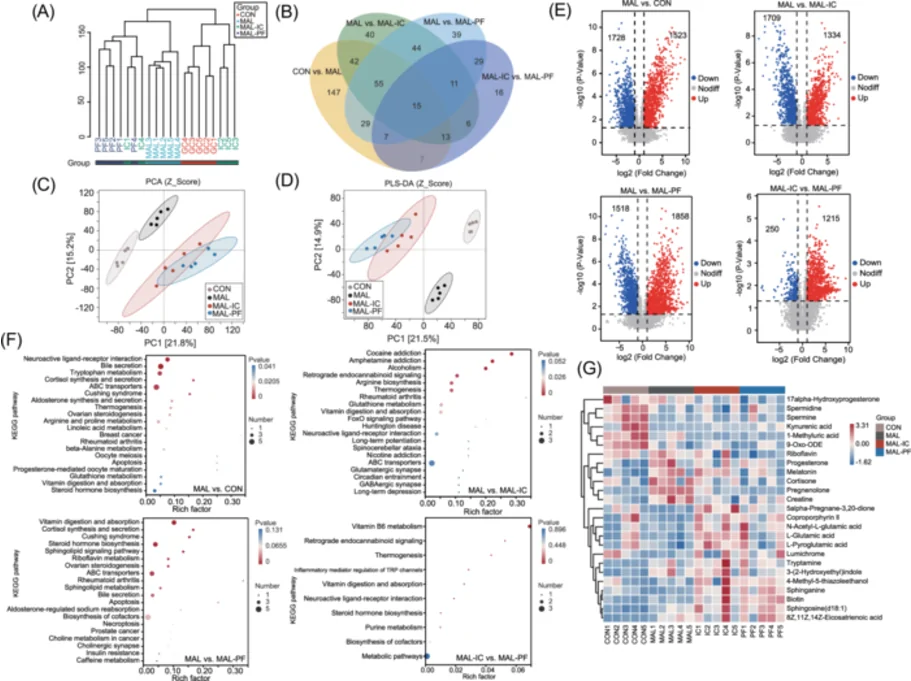
Pulse dietary intervention significantly shaped the untargeted metabolomics of the fecal samples. (A) The overall material clustering dendrogram, with colors representing different groups, suggests that metabolites are more similar as they cluster together. (B) The Venn diagram illustrates the outcomes of multiple pairwise comparison analyses, wherein the total count of differential metabolites for each comparison group is represented by the sum of numbers within each circle. The overlapping sections of the circles denote the shared differential metabolites between the two comparison groups. (C, D) PCA and PLS‐DA plots showing separation of metabolomic data as detected by the positive ion channel from CON (brown), MAL (black), MAL‐IC (red), and MAL‐PF (blue) mice. (E) Volcano plots illustrate the relative metabolite changes between pairwise comparisons among four groups of mice. Each point represents a metabolite. Red, blue, and gray dots indicate upregulated, downregulated, and non‐significant changes, respectively. (F) The enrichment results are evaluated through the Rich factor, *p*‐value, and the number of metabolites enriched in the pathway. The top 20 KEGG pathways with the smallest *p*‐value, which are the most significantly enriched, are selected for display: each point represents a metabolic pathway, with the horizontal axis showing the Rich factor and the vertical axis representing the enriched pathways. The dots represent the number of corresponding metabolic molecules on the pathway, with colors correlating to the *p*‐value. (G) Heatmap of differential metabolites demonstrates the correlation between the most representative and distinctive metabolites selected through volcano plots and enrichment factor plots across groups. Red denotes a positive correlation, whereas blue signifies a negative correlation. All data were obtained from the POS mode of mass spectrometry. Fecal samples were collected post‐intervention, with five samples per group subjected to untargeted metabolomic analysis (CON, mice fed a standard diet; MAL, mice fed a malnourished diet; MAL‐IC, mice fed a malnourished diet containing intact pulse cotyledon cells; MAL‐PF, mice fed a malnourished diet containing pulse flour).

Volcano plots further illustrated significant differences in pairwise comparisons, identifying numerous differentially expressed metabolites (Figure 5E, Figure S4). Differentially enriched metabolites were predominantly found in pathways such as arginine and proline metabolism, beta‐alanine metabolism, glutathione metabolism, and steroid hormone biosynthesis (Figure 5F). To visualize variations in metabolite expression across groups, a heatmap was generated (Figure 5G). Compared to the CON group, the MAL group exhibited lower levels of spermidine, spermine, kynurenic acid, and 1‐methyluric acid, and higher concentrations of cortisone, pregnenolone, and creatine. Notably, these metabolic changes appeared independent of growth and developmental processes. Further investigation revealed that the integrity of pulsed cells affected the expression of N‐acetyl‐L‐glutamine, L‐glutamate, and L‐pyroglutamic acid, with elevated levels observed exclusively in the MAL‐IC group compared to the MAL‐PF group.

### MAL‐IC diet promotes SCFA production, supporting systemic growth

To investigate how SCFAs promote growth by influencing the GH/IGF‐1 axis, we measured SCFA concentrations in fecal samples [24]. SCFA levels—specifically acetate, propionate, and butyrate—were markedly lower in the MAL group compared to the CON group (Figure 6A–C), aligning with findings from a previous study [16]. Notably, only the MAL‐IC diet, not the MAL‐PF diet, led to a significant increase in SCFA production relative to the MAL group (Figure 6A–D). This enhancement likely stemmed from the intact cell walls of pulsed cotyledon cells, which contain more RS1, a substrate readily fermented by colonic bacteria.

**Figure 6:**
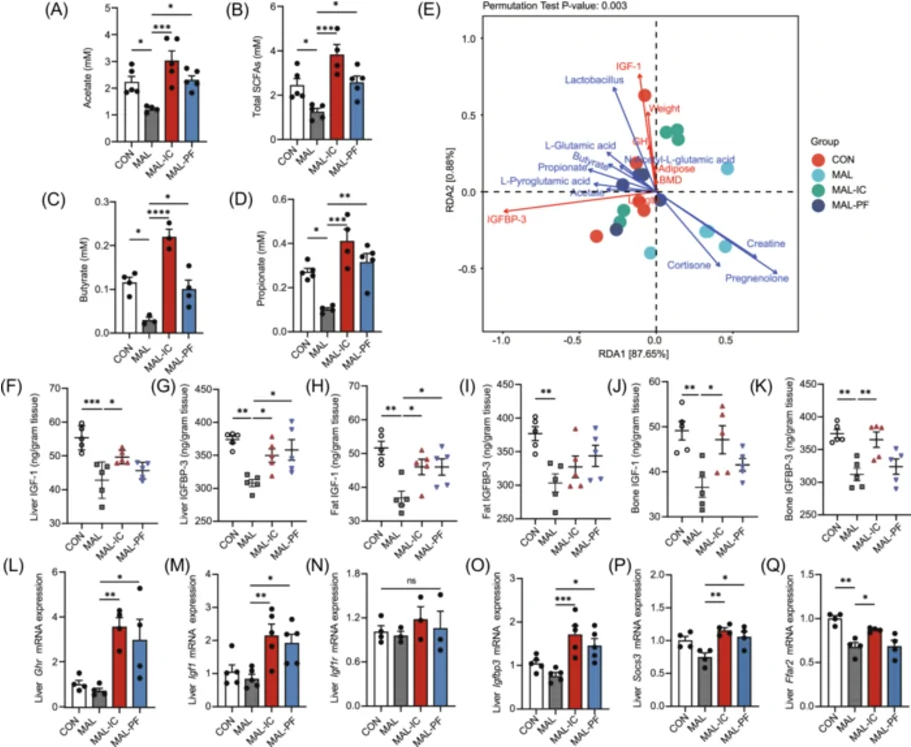
MAL‐IC diet promoted the production of SCFAs, and RDA analysis showed that SCFAs and *Lactobacillus* are important for growth. (A–D) The production of acetate, total SCFAs, butyrate, and propionate of CON, MAL, MAL‐IC, and MAL‐PF (*n* = 5 per group). (E) Redundancy analysis reveals the correlations among key growth indicators (body weight, body length, bone density, fat weight, GH, IGF‐1, IGFBP‐3), differential metabolites, and the abundances of *Lactobacillus*. Points of different colors represent samples within different groups, with red arrows indicating key growth indicators and blue arrows representing various influencing factors. The smaller the angle between the radial arrows, the greater the correlation. Additionally, the longer the length of the radial arrows, the greater the impact. MAL‐IC diet promoted systemic somatotropic axis activity in the MAL group. (F–K) IGF‐1 and IGFBP‐3 secretion levels in liver, fat, and bone tissues of CON, MAL, MAL‐IC, and MAL‐PF (*n* = 5 per group). The whiskers represent SEM. (L–Q) Expression levels of *Ghr*, *Igf*, *Igf1r*, *Igfbp3*, *Socs3*, and *Ffar2* in the liver tissue of CON, MAL, MAL‐IC, and MAL‐PF (*n* = 5 per group). Data are presented as mean ± SEM. *p < 0.05, **p < 0.01, ***p < 0.001, ****p < 0.0001; ns, not significant. (CON, mice fed a standard diet; MAL, mice fed a malnourished diet; MAL‐IC, mice fed a malnourished diet containing intact pulse cotyledon cells; MAL‐PF, mice fed a malnourished diet containing pulse flour).

We also performed redundancy analysis (RDA) to assess the relationships between growth indicators and key differential metabolites (Figure 6E). Results revealed that *Lactobacillus* abundance showed the strongest correlation with growth parameters, as evidenced by the minimal angle between its vector and those representing weight, length, adipose tissue mass, and BMD. Additionally, acetate, propionate, and butyrate levels were positively correlated with GH, IGF‐1, and systemic growth parameters (weight, length, adipose tissue, and BMD). *Lactobacillus* can ferment carbohydrates to produce substantial amounts of SCFAs [49]. Accordingly, we postulated that the therapeutic effects of the MAL‐IC diet on malnutrition in murine models are mediated through complex interactions between SCFAs and the somatotropic axis.

### MAL‐IC diet enhances somatotropic axis activity

The somatotropic axis is a critical endocrine regulatory network that primarily comprises GH, IGF‐1, and their associated receptors and binding proteins [52] (Figure 6F–Q). The liver is the principal site of IGF‐1 biosynthesis [53], releasing most IGF‐1 and IGFBP‐3 into systemic circulation, where they form extracellular complexes that facilitate IGF‐1 transport and mediate its physiological functions [54]. Additionally, IGF‐1 is produced by peripheral tissues, such as adipose tissue and bone, stimulating tissue growth via autocrine and paracrine mechanisms [55]. Quantification in the current study of IGF‐1 and IGFBP‐3 levels in the liver, fat, and bone tissues revealed significant reductions in the MAL group compared to the CON group (Figure 6F–K), consistent with our serum findings and previous studies [55] (Figure 3L–N). Notably, the MAL‐IC diet, but not the MAL‐PF diet, significantly increased these levels compared to the MAL group (Figure 6F–K). IGF‐1 promotes the synthesis of glucose, proteins, and fat, inhibits catabolism, stimulates RNA and DNA synthesis and cellular proliferation, and mediates the proliferative effects of GH in peripheral target organs and tissues [56, 57]. Thus, the beneficial effects of the MAL‐IC diet on IGF‐1 secretion may support growth in MAL mice.

Growth hormone receptors (GHRs) are predominantly expressed in the liver and serve as key indicators of GH secretion and tissue sensitivity [58]. Our analysis revealed significantly elevated *Ghr* expression in the MAL‐IC and MAL‐PF groups compared to the MAL group, with the MAL‐IC diet demonstrating more pronounced effects (Figure 6L). Notably, the MAL‐IC diet induced the synchronous upregulation of *Igf1* and *Igfbp3* (Figure 6M,O), consistent with previous findings [31]. Suppressors of cytokine signaling (SOCS) proteins, notably SOCS3, act as negative feedback regulators of IGF‐1 signaling [58]. While there was no marked difference in liver *Socs3* expression between the CON and MAL groups, undernourished mice showed a downward trend, suggesting a possible endocrine disorder. Meanwhile, MAL‐IC and MAL‐PF diets restored *Socs3* expression to normal levels (Figure 6P). Accumulating evidence indicates that SCFA supplementation enhances IGF‐1 production in malnourished mice and children [10, 24, 59, 60], a process mediated by free fatty acid receptors 2 and 3 (FFAR2/3) activated by SCFAs such as acetate, propionate, and butyrate [61]. Notably, the expression of FFAR2—the primary acetate receptor—was downregulated in the livers of malnourished mice; however, this effect was reversed by the MAL‐IC diet compared to the MAL‐PF diet (Figure 6Q, Figure S5). Together with the fecal SCFA analysis (Figure 6A–D), these results suggest that intact pulse cotyledon cell‐derived SCFAs produced by *Lactobacillus* enhance somatotropic axis activity via the gut–liver axis, thereby supporting systemic growth (Figure 7).

**Figure 7:**
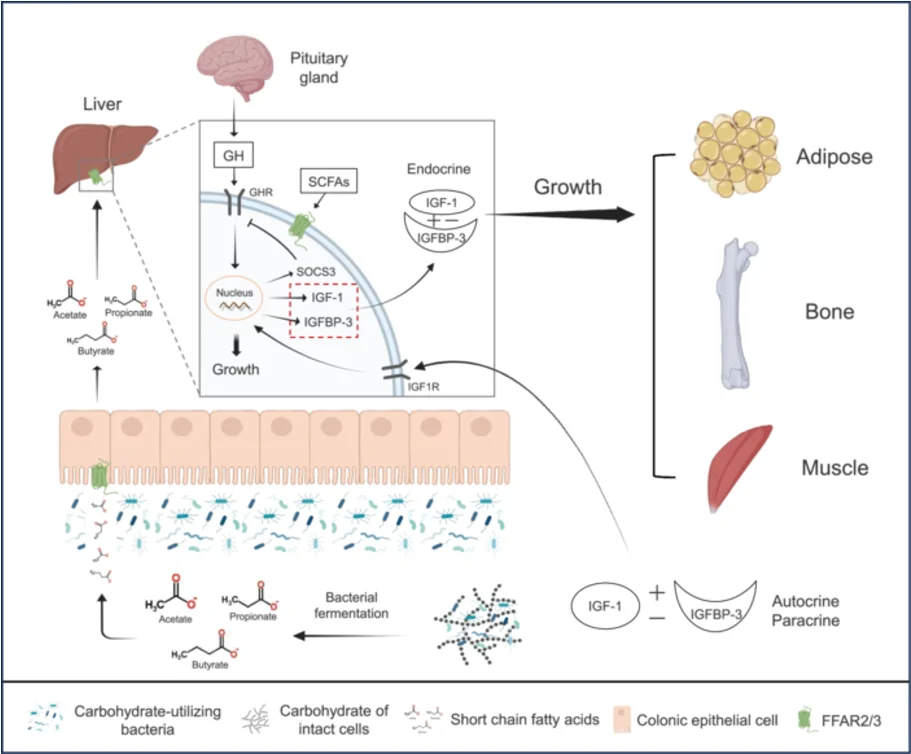
Potential mechanism of the MAL‐IC diet on systemic growth in MAL mice. The MAL‐IC diet may promote the growth process through the regulation of gut microbiota, particularly by increasing the abundance of carbohydrate‐utilizing bacteria by providing more RS1, which enhances the production of short‐chain fatty acids (SCFAs). These SCFAs circulate to the liver via the gut–liver axis, where they bind to the FFAR2 and activate the somatotropic axis. This activation stimulates GHR‐mediated GH signaling, leading to increased IGF‐1 and IGFBP‐3 production, ultimately promoting systemic growth, including adipose, bone, and muscle tissues. IGF‐1 and IGFBP‐3 can also be produced by peripheral tissues to activate the somatotropic axis via IGF1R. SOCS3 serves as a negative feedback regulator of IGF‐1 signaling. This diagram is created by Biorender. (CON, mice fed a standard diet; MAL, mice fed a malnourished diet; MAL‐IC, mice fed a malnourished diet containing intact pulse cotyledon cells; MAL‐PF, mice fed a malnourished diet containing pulse flour; RS1, type 1 resistant starch; FFAR2, free fatty acid receptor 2; GHR, growth hormone receptors; IGF‐1, insulin‐like growth factor‐1; IGFBP‐3, insulin‐like growth factor‐binding protein‐3; IGF1R, insulin‐like growth factor 1 receptor; SOCS3, suppressors of cytokine signaling proteins 3).

## 3 CONCLUSION

This study provides a systematic evaluation, demonstrating that intact pulse cotyledon cells are more effective than conventional pulse flour in mitigating malnutrition in mice. Mechanistically, the MAL‐IC diet enriches beneficial carbohydrate‐utilizing bacteria, such as *Lactobacillus* spp. and enhances the production of SCFAs, particularly acetate. These SCFAs circulate via the gut–liver axis, activating hepatic FFAR2 and subsequently stimulating the somatotropic axis. This process facilitates systemic growth by augmenting GHR‐mediated signaling and increasing IGF‐1 and IGFBP‐3 expression levels. Cumulatively, these results underscore the importance of maintaining cellular integrity in pulses to efficiently deliver nutrients to the colon and modulate gut microbiota composition, supporting an effective whole‐food approach for combating malnutrition.

## 4 MATERIALS AND METHODS

### Preparation of intact cell and pulse flour

White kidney beans (*Phaseolus vulgaris* L.) were purchased from a supermarket in Guangzhou, China. Figure S6 illustrates the preparation of intact white kidney bean cells based on the protocol detailed by Wu et al., with minor adaptations [17]. Briefly, pulse seeds were soaked in a 0.1 M Na_2_CO_3_ solution (1:5, w/v) for 16 h at 4°C [62]. After removing the seed coats, the hulled seeds were heated at 60°C for 30 min in the same soaking solution. The seeds were subsequently manually ground using a mortar and pestle, then thoroughly washed with distilled water through a series of sieves with mesh sizes of 150 and 54 μm. Material retained on the 54 μm size was collected and freeze‐dried. For pulse flour preparation, the heated seeds were blended (Midea Group, China) and then freeze‐dried (Figure S7). To simulate the baking process, both intact pulse cotyledon cells and pulse flour samples were baked in an oven at 180°C for 20 min [17].

### Animal experiments

Forty weaned C57BL/6J specific pathogen‐free mice (3‐week‐old) were purchased from Zhuhai BesTest Bio‐Tech Co., Ltd. After a 1‐week acclimation phase, the mice were randomly assigned to two groups: a CON group (*n* = 10) fed a standard diet (7% fat, 20% protein) for 8 weeks, and a MAL group (*n* = 30) fed a low‐fat, low‐protein diet (2% fat, 6.4% protein) for 4 weeks to induce moderate malnutrition as previously described [26]. After 4 weeks, the mice in the MAL group were subdivided into three groups: the MAL‐IC group was transitioned to a MAL diet containing 10% intact pulse cotyledon cells (MAL‐IC diet) for 4 weeks; the MAL‐PF group was transitioned to a MAL diet containing 10% pulse flour (MAL‐PF diet) for 4 weeks; the MAL group continued on the MAL diet for 4 weeks (Table S1; Figure 1A; Figure S8).

Body weight, body length, and food intake were recorded twice weekly. DEXA and oral glucose tolerance tests (OGTT) were performed at the end of Week 7. At the end of week 8, fecal samples were collected for 16S rRNA sequencing, untargeted metabolomic analysis, and SCFA measurement. After euthanasia, blood was collected for serum biochemical and cytokine assays; liver, fat, and bone tissues were harvested for further analysis (Figure S9).

### Dual‐energy X‐ray absorptiometry (DEXA)

DEXA procedures were conducted in accordance with established protocols [61]. At the end of week 7, BMD, BMC, BV, BA, adipose tissue mass, and lean tissue mass per unit scanned area were evaluated via DEXA (InAlyzer, Medikors Inc.).

### Oral glucose tolerance test (OGTT)

The OGTT was performed as described previously [63]. At the end of Week 7, the mice were fasted overnight, and glucose (2 g/kg body weight) was administered via gavage. Blood was sampled at baseline (0 min) and post‐administration intervals (30, 60, and 120 min) to assess glucose levels using a Yuwell 580 glucometer (Jiangsu Yuyue Medical Equipment Co., Ltd.). The AUC for glucose tolerance was calculated based on the blood glucose values at the four time points.

### Hematoxylin and eosin (H&E) staining

Liver samples were fixed in 4% paraformaldehyde, processed for paraffin embedding, dehydrated, and sectioned into thin slices (3–4 μm thickness). Sections were stained with H&E for histological assessment. Morphological features were analyzed using an Olympus BX‐51 light microscope (Olympus Corporation), and digital images were acquired with SlideViewer software (3D Histech Ltd.) for subsequent qualitative analysis.

### Serum biochemical parameter assessment

This assay was adapted from previous research with modifications [64]. Concentrations of key metabolic indicators—TG, CHOL, ALT, and AST—were quantified using a Hitachi 7180 automated biochemical analyzer (Hitachi High‐Technologies Corp.).

### Serum IL‐10, TNF‐α, IL‐1β, and IFN‐γ detection

Concentrations of IL‐10 (cat. no. ml037873), TNF‐α (cat. no. ml002095), IL‐1β (cat. no. ml106733), and IFN‐γ (cat. no. ml059749) in the serum were measured using the Mouse ELISA Kit (Shanghai Enzyme‐linked Biotechnology Co., Ltd.), following the manufacturer's instructions.

### 16S rRNA sequencing

Amplicon sequencing of 16S rRNA was conducted with slight modifications from a reported method [65]. Genomic DNA was isolated from mouse fecal samples using the Mag‐Bind® Soil DNA Kit (M5635‐02) (Omega Bio‐Tek, GA). DNA purity and concentration were determined before library construction. The V3–V4 region of the 16S rRNA gene was amplified using primers 338F (5'‐ACTCCTACGGGGGAGGCAGCA‐3') and 806R (5'‐GGACTACHVGGGGTWTCTAAT‐3'). The amplified PCR products were purified with Agencourt AMPure XP beads (Beckman Coulter, GA; cat. no. A63880). Sequencing was performed on an Illumina NovaSeq. 6000 sequencer (Illumina). Amplicon sequence variants were generated by filtering raw sequences using the DADA2 plugin in QIIME 2 version 2019.4 [66].

### Untargeted metabolomics analysis in feces

Untargeted metabolomic profiling was performed according to an established protocol with minor modifications [26]. Each mouse fecal sample was thawed at 4°C, blended with 1 mL of a pre‐chilled solvent solution comprising methanol, acetonitrile, and water (2:2:1, v/v), and then mixed. The mixture was vortexed and subjected to low‐temperature ultrasonic decomposition for 30 min. Subsequently, the samples were incubated at −20°C for 10 min to facilitate protein precipitation and centrifuged (14,000×*g*, 20 min, 4°C). The clarified supernatants were filtered and processed for liquid chromatography‐tandem mass spectrometry. Chromatographic separation was performed using an ultra‐high‐performance liquid chromatography system (1290 Infinity LC, Agilent Technologies) fitted with dual column configurations: hydrophilic interaction chromatography and reversed‐phase liquid chromatography columns. Mass detection was performed on a quadrupole time‐of‐flight analyzer (AB SCIEX TripleTOF 6600, MA), operated in both positive and negative electrospray ionization modes.

### Fecal SCFA determination

SCFA concentrations in mouse fecal samples were quantified using a previously described method with minor modifications [67]. Mouse feces (40 mg) were mixed with PBS (1:10 w/v) at 4°C. After centrifugation (12,000 rpm, 10 min), 240 μL of supernatant was collected. Subsequently, 60 μL of 15% metaphosphoric acid containing 50 mM 4‐methylpentanoic acid as an internal standard was added. SCFA identification and quantification were performed by gas chromatography with a flame ionization detector (GC‐FID, Agilent Technologies, Inc.) [64]. Chromatographic separation was performed on a polar column (Zebron, ZB‐FFAP, 30 m × 0.25 mm × 0.25 mm) under a constant nitrogen flow of 1 mL/min. The oven temperature was initially set at 60°C, ramped up to 220°C at 20°C/min, and held at 220°C for 3 min. Injector and detector temperatures were maintained at 280°C and 250°C, respectively. Target SCFAs in the samples were quantified by normalizing relative peak areas to the internal standards.

### Detection of IGF‐1, IGFBP‐3, and GH in liver, fat, bone, and serum samples

The concentrations IGF‐1 and IGFBP‐3 in liver, fat, bone, and serum samples were measured using a Mouse IGF‐1 ELISA Kit (cat. no. DCM186‐1), and a Mouse IGFBP‐3 ELISA Kit (Shanghai Enzyme‐Linked Biotechnology Co., Ltd.; cat. no. ml058183), according to the manufacturers' protocols. Serum GH levels were determined using the Mouse GH ELISA Kit (Shanghai Enzyme‐Linked Biotechnology Co., Ltd.; cat. no. ml063812), following the manufacturer's instructions.

### Real‐time quantitative PCR

Total RNA was extracted from liver tissue using the EZ‐10 Total RNA Mini‐Preps Kit (Shanghai Sangon Biotech Co., Ltd.), following the manufacturer's protocol. RNA purity and concentration were assessed using a NanoDrop spectrophotometer (Thermo Fisher Scientific). First‐strand cDNA synthesis was carried out using the MightyScript First Stand cDNA Synthesis Master Mix Kit (Shanghai Sangon Biotech Co., Ltd.) per the manufacturer's instructions. Primer pairs targeting genes were designed using PrimerBank (https://pga.mgh.harvard.edu/primerbank/) (Table S2). Quantitative real‐time PCR was performed with SYBR Premix Ex Taq II (Nanjing Vazyme Biotech Co., Ltd.) on a CFX384 Touch Real‐Time PCR Detection System (Bio‐Rad Laboratories). Cycle threshold values for each gene were normalized to the reference gene, glyceraldehyde‐3‐phosphate dehydrogenase (*Gapdh*).

### Statistical analysis and bioinformatics

All data were analyzed using GraphPad Prism v9.0 and are presented as mean ± standard error of the mean (SEM). The Shapiro–Wilk test was employed to evaluate the normality of data distribution, while the Brown–Forsythe test assessed the homogeneity of variances. For datasets that satisfied both normality and variance homogeneity assumptions, one‐way ANOVA followed by Tukey's post hoc test was conducted. In cases where these assumptions were not met (see Figures 1I, 2G,I, and 4A), the non‐parametric Kruskal–Wallis test with Dunn's post‐hoc analysis for pairwise comparisons was applied. Statistical significance was defined as a *p*‐value < 0.05.

Gut microbiota analysis included assessments of α‐diversity metrics (Chao1, Observed OTUs, and Shannon indices) and β‐diversity (Bray–Curtis and Unifrac‐unweighted distances via PCoA) using 16S rRNA sequencing data. Differentially abundant taxa were identified using LEfSe and random forest algorithms, and their significance for group differentiation was evaluated.

For untargeted metabolomic profiling, PCA and PLS‐DA were performed, with differential metabolites identified using volcano plots, and enriched KEGG pathways were investigated. Representative metabolite expression patterns across groups were displayed via heatmaps.

RDA incorporated variables including growth parameters (body weight, body length, adipose mass, and BMD), hormone levels (GH, IGF‐1, and IGFBP‐3), SCFAs, and microbial abundance (e.g., *Lactobacillus*). All microbiome and metabolome analyses were performed using GenesCloud (Personal Gene Cloud, https://www.genescloud.cn), a comprehensive bioinformatics platform.

## AUTHOR CONTRIBUTIONS

**Yumeng Yan**: Conceptualization; investigation; methodology; software; data curation; visualization; formal analysis; writing—original draft. **Yumeng Yan, Zhuqing Xie, Zhipeng Gu, Zhongxin Li, Qiang Huang,** and **Bin Zhang**: Writing—reviewing & editing. **Qiang Huang** and **Bin Zhang**: Supervision. **Bin Zhang**: Funding acquisition; project administration. All authors have read the final manuscript and approved it for publication.

## CONFLICT OF INTEREST STATEMENT

The authors declare no conflicts of interest.

## ETHICS STATEMENT

The experimental procedures were approved by the Ethics Committee of Guangdong Academy of Sciences (approval no. K202401191489).

## Supporting information


**Figure S1.** Bone mineral density (BMC), bone mineral content (BMC), bone volume and bone area of the mouse femur.
**Figure S2.** Relative abundance of bacteria by family classification.
**Figure S3.** Relative abundance of *Lactobacillus*.
**Figure S4.** Top 10 metabolites identified in all samples.
**Figure S5.** Free Fatty Acid Receptor 3 (*Ffar3*) mRNA expression levels in the liver. ns, not significant.
**Figure S6.** The preparation process of pulse flour and intact pulse cotyledon cells.
**Figure S7.** Bright‐field and polarized light microscopic images of IC and PF before and after dry heat treatment.
**Figure S8.** A schematic of the components of each diet, represented by the percentage of total calories (A) and the quality (B).
**Figure S9.** Experimental process of animal test.

**Table S1.** Composition of control diet and malnourished diet.
**Table S2.** Primer sequences.

## Data Availability

All data supporting the findings of this study are available in the manuscript or Supplementary Data. The data used are saved in GitHub https://github.com/g834824545/20251212iMetaOmics. The 16S rRNA sequencing data generated in this study are publicly accessible in the NCBI Sequence Read Archive (SRA) under the accession code PRJNA1366738 (https://www.ncbi.nlm.nih.gov/bioproject/PRJNA1366738). Supplementary materials (figures, tables, graphical abstract, slides, videos, Chinese translated version, and update materials) may be found in the online DOI or iMeta Science http://www.imeta.science/imetaomics/.
