# Supplementary material for: Intact pulse cotyledon cell diet improves systemic growth via the somatotropic axis in malnourished mice
Source: IMetaOmics. 2026 Feb 25;3(1):e70085. doi: 10.1002/imo2.70085 (PMC13674392; doi:10.1002/imo2.70085)
Supplement: Supplementary file 1 [file IMO2-3-e70085-s001.docx]

**Supplementary material for**

**Intact pulse cotyledon cell diet improves systemic growth via the somatotropic axis in malnourished mice**

Running title: Pulse cell diet improves growth

Yumeng Yan^1^, Zhuqing Xie^1^, Zhipeng Gu^1^, Zhongxin Li^1^, Qiang Huang^1^, Bin Zhang^1^

^1^ School of Food Science and Engineering, National Engineering Research Center of Wheat and Corn Further Processing, Guangdong Province Key Laboratory for Green Processing of Natural Products and Product Safety, South China University of Technology, Guangzhou 510640, China

**Correspondence**

Bin Zhang.

Email: [zhangb@scut.edu.cn](mailto:zhangb@scut.edu.cn)

**Figure S1.** Bone mineral density (BMC), bone mineral content (BMC), bone volume and bone area of the mouse femur. * *p* < 0.05, ** *p* < 0.01. (CON, mice fed a standard diet; MAL, mice fed a malnourished diet; MAL-IC, mice fed a malnourished diet containing intact pulse cotyledon cells; MAL-PF, mice fed a malnourished diet containing pulse flour)

**Figure S2.** Relative abundance of bacteria by family classification. (CON, mice fed a standard diet; MAL, mice fed a malnourished diet; MAL-IC, mice fed a malnourished diet containing intact pulse cotyledon cells; MAL-PF, mice fed a malnourished diet containing pulse flour)

**Figure S3.** Relative abundance of *Lactobacillus*. no significance, * *p* < 0.05. (CON, mice fed a standard diet; MAL, mice fed a malnourished diet; MAL-IC, mice fed a malnourished diet containing intact pulse cotyledon cells; MAL-PF, mice fed a malnourished diet containing pulse flour)

**Figure S4.** Top 10 metabolites identified in all samples.

**Figure S5.** Free Fatty Acid Receptor 3 (*Ffar3*) mRNA expression levels in the liver. no significance. (CON, mice fed a standard diet; MAL, mice fed a malnourished diet; MAL-IC, mice fed a malnourished diet containing intact pulse cotyledon cells; MAL-PF, mice fed a malnourished diet containing pulse flour)

**Figure S6.** The preparation process of pulse flour and intact pulse cotyledon cells.

**Figure S7.** Bright-field and polarized light microscopic images of IC and PF before and after dry heat treatment. (IC, intact pulse cotyledon cells; PF, pulse flour; DHT, dry heat treatment).

**Figure S8.** A schematic of the components of each diet, represented by the percentage of total calories (A) and the quality (B).

**Figure S9.** Experimental process of animal test.
